# Off-label use of dalbavancin in children: a case series

**DOI:** 10.1093/jac/dkae212

**Published:** 2024-07-03

**Authors:** Anna Gamell, Eneritz Velasco-Arnaiz, Maria Goretti López-Ramos, María Ríos-Barnés, Sílvia Simó-Nebot, Victoria Fumadó, Antoni Noguera-Julián, Clàudia Fortuny

**Affiliations:** Infectious Diseases Department, Hospital Sant Joan de Déu, Barcelona, Spain; Infectious Diseases Department, Hospital Sant Joan de Déu, Barcelona, Spain; Pharmacy Department, Hospital Sant Joan de Déu, Barcelona, Spain; Infectious Diseases Department, Hospital Sant Joan de Déu, Barcelona, Spain; Infectious Diseases Department, Hospital Sant Joan de Déu, Barcelona, Spain; CIBER de Epidemiología y Salud Pública (CIBERESP), Madrid, Spain; Infectious Diseases Department, Hospital Sant Joan de Déu, Barcelona, Spain; CIBER de Epidemiología y Salud Pública (CIBERESP), Madrid, Spain; Department of Paediatrics, University of Barcelona, Barcelona, Spain; Infectious Diseases Department, Hospital Sant Joan de Déu, Barcelona, Spain; CIBER de Epidemiología y Salud Pública (CIBERESP), Madrid, Spain; Department of Paediatrics, University of Barcelona, Barcelona, Spain; Red de Investigación Translacional en Infectología Pediátrica (RITIP), Madrid, Spain; Infectious Diseases Department, Hospital Sant Joan de Déu, Barcelona, Spain; CIBER de Epidemiología y Salud Pública (CIBERESP), Madrid, Spain; Department of Paediatrics, University of Barcelona, Barcelona, Spain; Red de Investigación Translacional en Infectología Pediátrica (RITIP), Madrid, Spain

## Abstract

**Introduction:**

Dalbavancin is an antibiotic active against most Gram-positive bacteria approved for acute bacterial skin and skin structure infections (ABSSSI). Owing to its long half-life, it is being increasingly used for other indications.

**Patients and methods:**

We present a case series of children and adolescents treated with dalbavancin for osteoarticular, catheter-related and other non-ABSSSI infections.

**Results:**

Dalbavancin was prescribed to 15 patients. Six (40%) were female and median age at prescription was 11.9 (IQR 1.3–18.0) years. Most of them (12/15) had significant comorbidities. Patients presented mainly with deep surgical site infections, osteoarticular infections and central-line-associated bloodstream infections. The most common isolate was *Staphylococcus aureus* followed by *Staphylococcus epidermidis.* Major reasons to prescribe dalbavancin were to ensure compliance and patients’ convenience. Two patients discontinued the drug due to adverse events possibly related to it. The rest of the patients completed the treatment with dalbavancin, with a median duration of 56 days (IQR 17.5, 115.5). All achieved complete resolution and present no relapse after a median follow-up of 9.9 months (IQR 4.8, 16.6).

**Conclusions:**

Dalbavancin was a safe, effective and convenient alternative in selected paediatric patients with complicated non-ABSSSI infections caused by Gram-positive bacteria.

## Introduction

Dalbavancin is a lipoglycopeptide antibiotic active against most Gram-positive bacteria. It has a unique pharmacokinetics (PK) with a terminal half-life of 14.4 days.^1^ Dalbavancin is approved as intravenous infusion for the treatment of acute bacterial skin and skin structure infection (ABSSSI) in adults and children 3 months and older by the European Medicines Agency^2^ and from birth by the US Food and Drug Administration.^3^ The safety and efficacy of dalbavancin in adults have been demonstrated in pivotal phase 3 trials^4^ and the recent approval in paediatrics is based on the results of PK studies and a phase 3 paediatric study in patients with ABSSSI.^5^

However, given its long-acting activity, prolonged concentrations in bone^6^ and convenience of administration, dalbavancin is increasingly being used for off-label indications, including osteoarticular, cardiovascular and catheter-related infections.^7,8^

We present a case series of children and adolescents treated with dalbavancin for non-ABSSSI infections caused by Gram-positive pathogens.

## Patients and methods

This retrospective observational study includes children and adolescents (≤18 years) treated with at least one dose of dalbavancin between 2020 and 2023 at Sant Joan de Déu Hospital, a 268 paediatric-bed tertiary-care maternal and children’s hospital in Barcelona, Spain. Decisions about the antimicrobial treatment were made on the basis of the bacterial isolate, the site of infection and the characteristics of each patient.

During admission and at each outpatient visit, clinical, laboratory and pharmacy data were collected electronically, using the data collection system of the hospital. Informed consent was obtained from all parents or guardians of the patients included in the study. This study was approved by the local Ethics Committee (code: ART-05-23).

## Results

### Clinical characteristics

We included 15 patients [six females, median age (range): 11.9 (0.3–18.9) years], most of them (12/15) affected with significant comorbidities (Table 1).

**Table 1.** Clinical characteristics of 15 children and adolescents treated with dalbavancin for infections other than ABSSSI

Patient	Sex; age at DAL prescription (years)	Underlying disease	Infection	Surgery	Aetiology	Previous antibiotic	Previous antibiotic duration (days)	Reason for DAL use	Dalbavancin doses	DAL duration (days)	Concomitant antimicrobial therapy	Follow-up since last dose (months)^a^	Outcome
#1	M; 0.32	Neonatal debut of Peutz-Jeghers syndrome requiring subtotal colectomy	CLABSI	no	*S. haemolyticus*	Vancomycin	2	No durable venous access	22.5 mg/kg on day 1 (single dose)	14	no	0.8	Cured
#2	F; 18.9	Idiopathic scoliosis	Postoperative spinal implant infection	Surgical debridement twice and 1 stage complete replacement of the implant	MSSA	Cefazolin plus linezolid, cloxacillin plus daptomycin, cotrimoxazole	21	Patient convenience, compliance	1.5 g on days 1 and 8 1 g × 11 every other week from day 28	183	Cotrimoxazole^b^	17.8	Cured
#3	F; 13.3	Rett syndrome, neuromuscular scoliosis	Postoperative spinal implant infection	2 stage partial replacement of the implant	MSSA	Levofloxacin plus rifampicin, daptomycin plus cefazolin; levofloxacin	44	Patient convenience, compliance	18 mg/kg on days 1 and 8 12 mg/kg × 2 every other week from day 28	56	no	21.1	Cured
#4	F; 18.7	Cerebral palsy, neuromuscular scoliosis	Postoperative spinal implant infection	1 stage partial replacement of the implant	CoNS	Vancomycin, levofloxacin plus rifampicin, daptomycin	21	Patient convenience, compliance	1.5 g on days 1 and 8 1 g × 3 every other week from day 28	69	Rifampicin^c^	12.7	Cured
#5	M; 6.2	Autism Spectrum Disorder, QT prolongation	chronic infection of a Gore-Tex patch used to repair a CDH	Prosthetic patch and costal arches (9 and 10) removal	MRSA	Vancomycin	4	No oral choice available	18 mg/kg on days 1 and 8 12 mg/kg × 7 every other week from day 28	119	no	13.8	Cured
#6	F; 1.1	Short bowel syndrome secondary to gastroschisis	CLABSI	no	MSSA	Vancomycin, cloxacillin, teicoplanin	19	Failure with other antibiotics	15 mg/kg on day 1 7.5 mg/kg on day 8	21	no	47.1	Cured
#7	F; 17.9	Dermatomyositis (under treatment with methotrexate and prednisone)	Chronic cellulitis	no	MSSA	Cefadroxil	18	Patient convenience, compliance	1 g on day 1 500 mg × 15 weekly from day 8	112	no	15.4	Cured
#8	M; 2.9	Acute lymphoblastic leukaemia	Spondylodiscitis	no	*S. epidermidis*	Daptomycin, linezolid	35	Patient convenience	22.5 mg/kg on days 1 and 8	21	no	9.9	Cured
#9	M; 11.9	None	Subacute hip osteomyelitis with pyomyositis^d^	Surgical debridement twice	MSSA	1st regimen: cefazolin, cefadroxil. Relapse: cefazolin plus clindamycin, cefazolin plus daptomycin	1st regimen: 42 Relapse: 7	Patient convenience	18 mg/kg on days 1 and 8 12 mg/kg × 9 every other week from day 28	152	no	4.5	Cured
#10	M; 4.9	Congenital heart disease (hypoplastic left heart syndrome)	Septic thrombophlebitis	Thrombectomy	*S. epidermidis*	Vancomycin, daptomycin	18	No durable venous access	22.5 mg/kg on day 1, then stopped due to AE (rash)	14	no		Cured (with linezolid)
#11	M; 0.63	Nephrogenic diabetes insipidus	CLABSI	no	*S. epidermidis*	Teicoplanin	2	No durable venous access	22.5 mg/kg on day 1 (single dose)	14	no	7.4	Cured
#12	F; 15.3	Humerus hypoplasia	Humerus osteomyelitis associated with external fixation	no	MSSA	Cefazolin	7	Patient convenience, compliance	1.5 g on days 1 and 8, then stopped due to AE (vomiting)	28	rifampicin		Cured (with cefadroxil)
#13	F; 1.3	Wolman disease	CLABSI	no	*S. epidermidis*	Vancomycin	2	No durable venous access	22.5 mg/kg on day 1 (single dose)	14	no	5.1	Cured
#14	M; 15.8	Myelomeningocele	Femur chronic osteomyelitis associated to stage 4 pressure-induced injury	yes	MRSA	Vancomycin, ceftaroline, daptomycin	5	Patient convenience	18 mg/kg on days 1 and 8 12 mg/kg × 3 every other week from day 28	70	no	1.0	Cured
#15	M; 18.2	None	Femur infection associated to fracture fixation device	yes	*S. lugdunensis*	Cefazolin	5	Patient convenience, compliance	1.5 g on days 1 and 8 1 g × 2 every other week from day 28	56	no	5.2	Cured

DAL, dalbavancin; MSSA, methicillin-sensible *Staphylococcus aureus*; CoNS, coagulase-negative staphylococci; MRSA, methicillin-resistant *Staphylococcus aureus*; AE, adverse event.

^a^Follow-up time not calculated for patients #10 and #12, as they did not finish the treatment with dalbavancin.

^b^Associated with DAL during the firsts 12 weeks of treatment.

^c^Associated with DAL for 12 days. Rifampicin was stopped due to toxicoderma.

^d^The child was initially treated for 6 weeks: 3 weeks after completing the antibiotic regimen, he had a relapse requiring surgical intervention.

Three patients (#2, #3 and #4) presented with deep surgical site infections after scoliosis corrective surgery. Five patients presented with osteoarticular infections: patient #8 had spondylodiscitis, #9 had complicated hip hematogenous osteomyelitis with pyomyositis, #12 had humerus osteomyelitis associated to external fixation, #14 had chronic osteomyelitis associated to stage 4 pressure-induced skin and soft tissue injury and #15 had late onset infection of the femur associated to a fracture fixation device. Four children had central-line-associated bloodstream infections (CLABSI) (#1, #6, #11 and #13). The remaining patients presented with chronic infection of a prosthetic patch used to repair a congenital diaphragmatic hernia (#5), chronic cellulitis on top of a calcinosis plaque secondary to dermatomyositis (#7) and septic thrombophlebitis of a cavopulmonary shunt (#10). Eight patients required surgery as part of the treatment of the infection. All patients had a bacterial isolate; two out of 15 had polimicrobial infections with Gram-negative bacillus (#4 and #14). The most common isolate was *Staphylococcus aureus* (eight out of 15; two methicillin-resistant), followed by *Staphylococcus epidermidis* (four out of 15) and other *Staphylococcus plasmocoagulase*-negative (three out of 12).

### Reasons to prescribe dalbavancin

In all cases, dalbavancin was prescribed as sequential therapy. Median time from antibiotic initiation to dalbavancin prescription was 7 days (IQR 4, 21). Most children in this case series required prolonged antibiotic treatment due to the nature of the infection (patient #8), presence of orthopaedic material (#2, #3, #4 and #12) or deep subacute or chronic infections (#5, #7, #9, #14 and #15). Thus, the main reasons for prescribing dalbavancin were to ensure compliance and patient convenience. Other reasons included failure to clear the infection with other antibiotics (#6), difficulty in obtaining a durable venous access (#1, #10, #11 and #13) or lack of oral antibiotic alternatives (#5).

### Dalbavancin treatment

The four patients with CLABSI received a single complete dose of dalbavancin (patient #6 received 15 mg/kg on day 1 and 7.5 mg/kg on day 8; while patients #1, #11 and #13 received a unique administration of 22.5 mg/kg). One adolescent patient (#7) received 1000 mg as an initial dose, followed by 15 500 mg doses on a weekly basis. Patient #10 received just one dose of 22.5 mg/kg, after which the treatment was discontinued due to a possible adverse effect. The remaining nine patients received two initial equal doses (22.5 mg/kg, 18 mg/kg or 1500 mg, depending on the age) on days 1 and 8, followed by maintenance doses every other week initiated on day 28 (15 mg/kg, 12 mg/kg or 1000 mg, depending on the age), except for patient #12, whose treatment was stopped before maintenance due to adverse effects. Patients were discharged after the first dose of dalbavancin and subsequent ones were administered in the day hospital.

Accounting for the long half-life of dalbavancin and the length of its activity, we calculated the duration of treatment as the days between the first and last dose plus 14 days. Using this approach, the median total dalbavancin duration for patients who completed the antibiotic course with this antibiotic was 56 days (IQR 17.5, 115.5). Total dalbavancin doses ranged from 1 to 16.

### Outcome

Two patients presented adverse events that led to the discontinuation of dalbavancin. Patient #10 presented fever and a diffuse confluent erythematous maculopapular rash, mostly affecting the trunk and without mucosal involvement 24 hours after the first administration of dalbavancin. He was also receiving captopril, diuretics, phenytoin, sedative medication, micafungin and ciprofloxacin. No more doses of dalbavancin were administered and he completed the treatment with linezolid. The patient did not need corticosteroids or antihistamines and the rash resolved within 36 hours. Patient #12 presented with one vomiting session after the first dose of the drug and several vomiting sessions, watery stools and abdominal discomfort following the second dose. We changed the antibiotic to oral cefadroxil.

The remaining 13 patients completed the treatment with dalbavancin and have been followed up for a median of 9.9 months (IQR 4.8, 16.6) since treatment completion. All of them achieved complete resolution of the infection and have had no recurrence.

## Discussion

This case series describes a real-life experience using dalbavancin in paediatric patients with non-ABSSSI Gram-positive bacterial infections who, in most cases, needed a prolonged antibiotic duration. To date, this is one of the first reports of children treated with dalbavancin for non-ABSSSI infections. In their recent publication, Caselli *et al*.^9^ present a paediatric case series with 31 patients who received treatment with dalbavancin. Among these, 19 had ABSSSI, while 12 presented with bone infections. They treated 15 patients with multiple doses. Specifically, nine patients received two doses, four patients received three doses, one patient received six doses and another patient received nine doses. Notably, there were no dose adjustments following the initial administration.

Dalbavancin is approved solely to treat ABSSSI. However, several reports in adults have documented that it is safe and effective for the treatment of prosthetic joint infections, catheter-related bloodstream infections and endocarditis, even in prolonged courses.^10–12^

Most patients in our series were affected with significant comorbidities and presented with Gram-positive infections that required prolonged antibiotic regimens and often surgical treatments, representing a major challenge for adherence to antibiotics. In this context, dalbavancin allowed treatment simplification in terms of earlier hospital discharge and reduction of intravascular catheter manipulations and days.

While dalbavancin was used as sequential therapy in most cases, it also proved useful in one patient with failure of previous treatments (#6). Also, there was no oral antibiotic alternative for patient #5. Although there were oral antibiotic options for the rest of the patients, the pill burden, the age of the child and some of the comorbidities challenged the compliance with oral treatment. Thus, in our experience, the main reasons to prescribe dalbavancin were to ensure adherence and patient convenience.

According to PK studies in the paediatric population, dalbavancin approved dosing is 22.5 mg/kg as a single dose in infants and children from birth to 6 years and 18 mg/kg as a single dose in children and adolescents from 6 to 18 years, with a maximum dose of 1500 mg.^2,3,13,14^ To date, there have been no published studies on multiple dosing of dalbavancin for subacute or chronic infections in children. In adults, different schedules have been used for multiple dosing.^15^ Recent PK studies show a 4–6-week adequate antibiotic coverage after the administration of two doses of 1500 mg 1 week apart.^16,17^ It is unknown whether this PK behaviour is applicable in children. Cojutti *et al.* recently showed creatinine clearance as a covariant in dalbavancin clearance in adult patients.^17^ By the age of 12–24 months, the glomerular filtration rate reaches young adult values. Thus, creatinine clearance in children without renal disease usually exceeds 90 mL/min/1.73 m^2^.^18^ All patients had a normal estimated glomerular filtration rate calculated with the revised Schwartz equation. Therefore, we decided to use a dalbavancin schedule of two initial doses on days 1 and 8 followed by maintenance doses from day 28 (see Table 2). For maintenance dosing we mostly prescribed the schedule that uses double doses every other week to simplify the regimen and improve compliance, as previously reported in adults.^15^

**Table 2.** Summary of multi-dose regimens used in our series

Schedule	Patients	Initial dose	Maintenance doses
Schedule 1: weekly doses for maintenance	≥6 years (patients #7)	12 mg/kg (maximum 1000 mg) day 1	7.5 mg/kg (maximum 1000 mg) day 8 and every 7 days
Schedule 2: double dose every other week for maintenance	≥6 years (patients #2, #3, #4, #5, #9, #14 and #15)	18 mg/kg (maximum 1500 mg) day 1 and 8	12 mg/kg (maximum 1000 mg) day 28 and every 14 days

Dalbavancin was well tolerated in most cases. Only two patients presented adverse reactions possibly related to the drug, which led to treatment discontinuation. Recent reviews and meta-analysis report that dalbavancin has a comparable safety profile to other antibiotics frequently used to treat Gram-positive infections.^19^ Skin rash and nausea and vomiting are described among the most common adverse events related to dalbavancin, occurring in approximately 2%–3% of patients.^19,20^

Treatment with dalbavancin was successful in all patients who completed the course, and none presented with relapse of the infection after a median follow-up of 9.9 months.

This study has limitations. It reports a single centre experience, patients presented with a heterogeneity of comorbidities, we did not specifically test dalbavancin susceptibility of the isolates and we used different dosing regimens. Moreover, we did not use a survey to measure our patients’ satisfaction. However, when offered the switch to dalbavancin, parents of young children appreciated having an alternative to the multi-dose daily administration of an oral antibiotic, and adolescents expressed their preference to attend the hospital once every 1 or 2 weeks to receive dalbavancin over daily oral treatments. Adherence to oral antibiotics after hospital discharge is described to be low with poor clinical outcomes.^21^ Treatment compliance is key to reach the goal of curation and strategies that contribute to ensure optimal adherence are most needed, especially among young children and adolescents.

### Conclusions

In our experience, dalbavancin was a safe, effective and convenient alternative in selected paediatric patients with complicated non-ABSSSI infections caused by Gram-positive bacteria. Dalbavancin contributed to ensure compliance and allowed a reduction in the length of hospital stay and catheter manipulation.
